# Supplementary material for: From calibration to parameter learning: Harnessing the scaling effects of big data in geoscientific modeling
Source: Nat Commun. 2021 Oct 13;12:5988. doi: 10.1038/s41467-021-26107-z (PMC8514470; doi:10.1038/s41467-021-26107-z)
Supplement: Supplementary file 1 — Supplementary Information [file 41467_2021_26107_MOESM1_ESM.pdf]

Supplementary Information for

From calibration to parameter learning: Harnessing the scaling effects of big data in geoscientific modeling

Nature Communications

Wen-Ping Tsai[1], Dapeng Feng[1], Ming Pan[2,3], Hylke Beck[4], Kathryn Lawson[1,5], Yuan Yang[6,7], Jiangtao Liu[1], and Chaopeng Shen[1,5]*

[1] Civil and Environmental Engineering, Pennsylvania State University, University Park, PA, USA
[2] Center for Western Weather and Water Extremes, Scripps Institution of Oceanography, University of California San Diego, La Jolla, CA, USA
[3] Civil and Environmental Engineering, Princeton University, Princeton, NJ, USA
[4] GloH2O, Almere, the Netherlands
[5] HydroSapient, Inc., State College, PA, USA.
[6] Department of Hydraulic Engineering, Tsinghua University, Beijing, China
[7] Institute of Science and Technology, China Three Gorges Corporation, Beijing, China

* Corresponding author: Chaopeng Shen. Email: cshen@engr.psu.edu

Content of this file

Figures S1-S6

Table S1

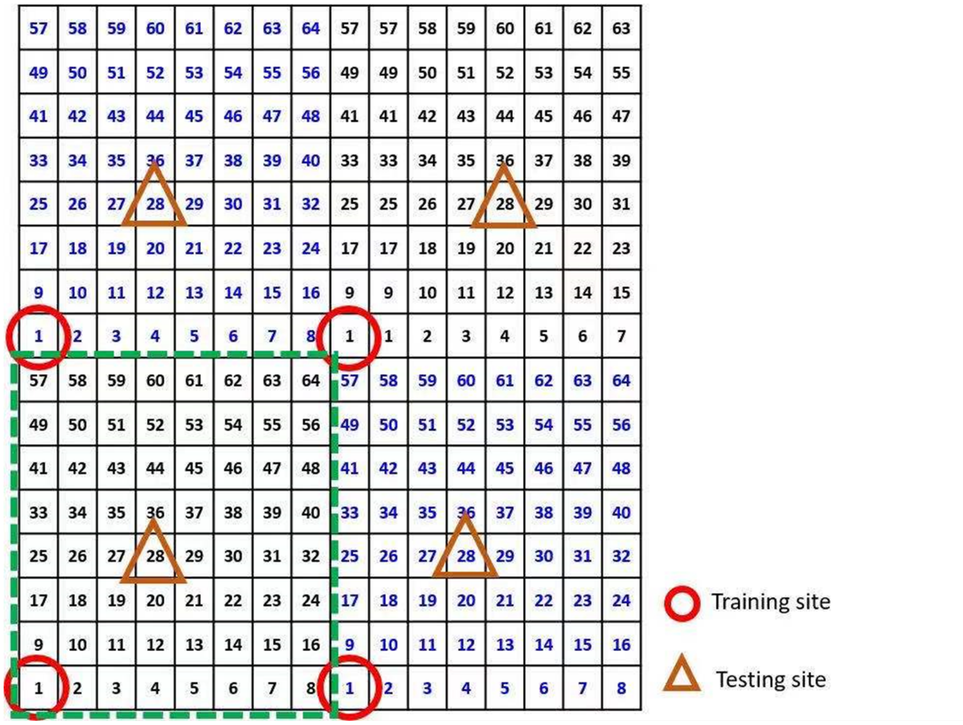

Figure S1. *An illustration of the sampling strategy and the training and test sites at density s8. The green dashed line indicates one 8*8 patch (64 gridcells).*

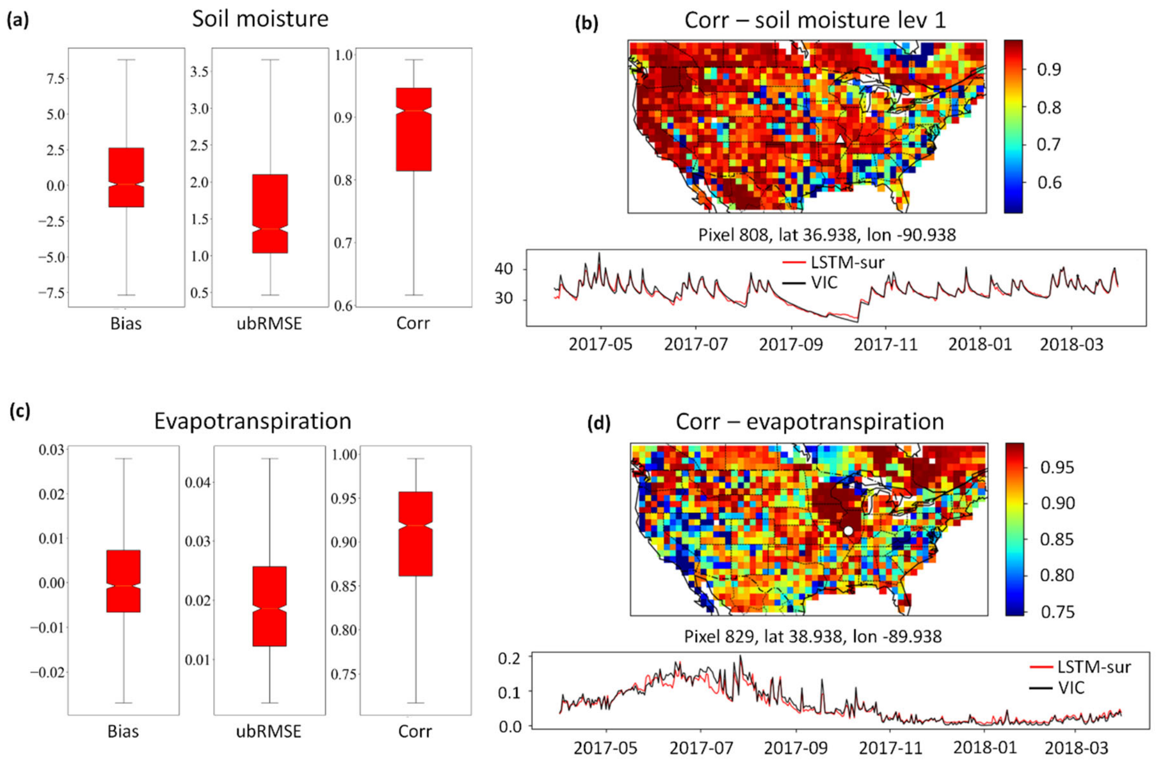

Figure S2. *The performance of the LSTM surrogate model (LSTM-sur). (a) Test metrics for the soil moisture of LSTM-sur. (b) Map of correlation between soil moisture from LSTM-sur and VI, and time series comparison at a randomly chosen gridcell (white triangle). Black line denotes simulation of LSTM-sur. Red line denotes VIC. (c) Test metrics for the evapotranspiration of LSTM-sur as compared to VIC outputs. (d) Map of correlation between evapotranspiration from LSTM-sur and VIC; and time series comparisons at a randomly chosen gridcell (white circle) for evapotranspiration. The boxplots' lower whisker, lower box edge, middle line, upper box edge and upper whisker represent 10th, 25th, 50th, 75th, and 90th percentiles, respectively.*

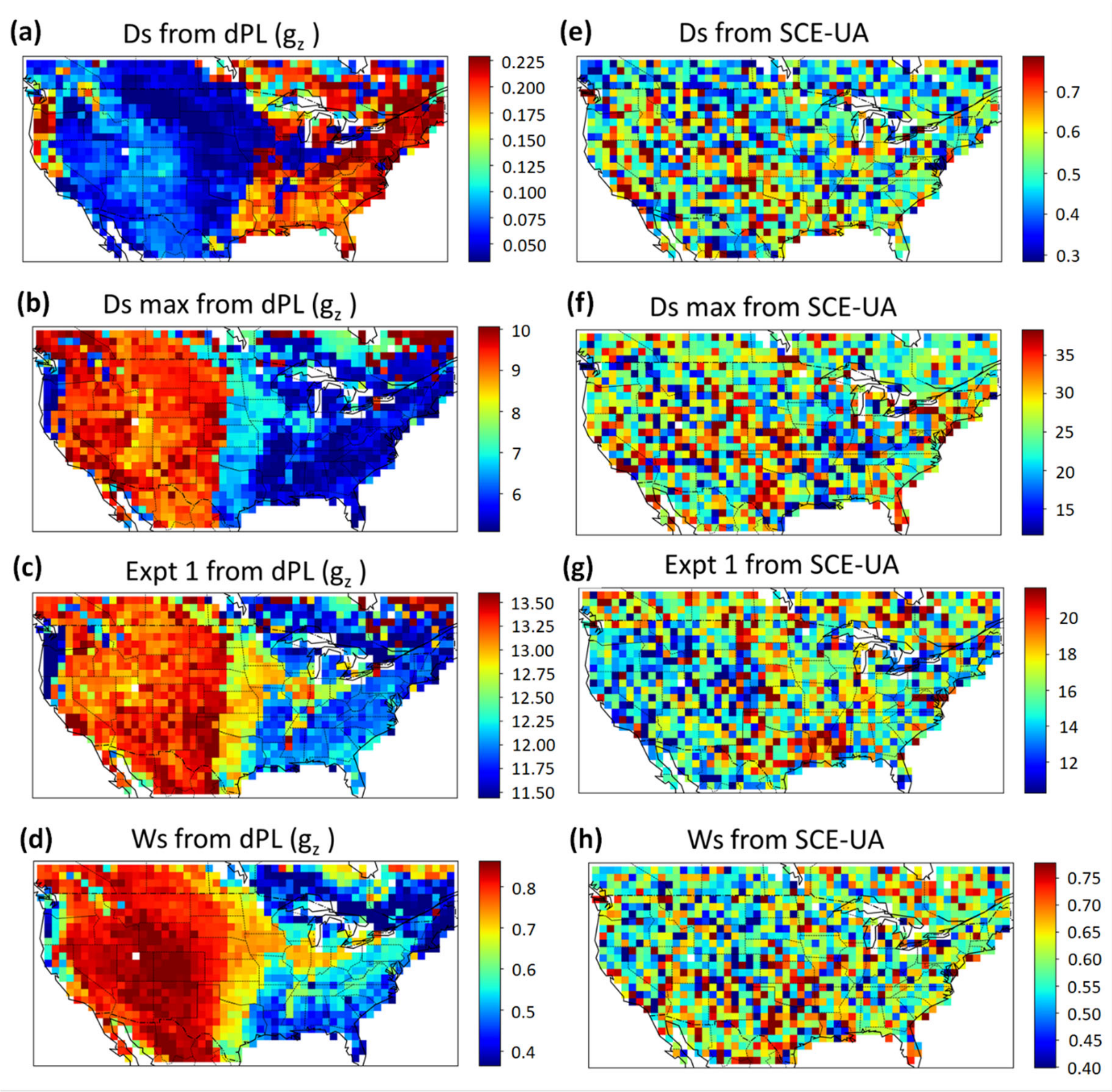

Figure S3. *Maps of parameters other than INFILT. Except for Ds max (unit: mm/day), other parameters are unitless. Explanations for the variables are provided in the "parameter estimation network" section in the main text.*

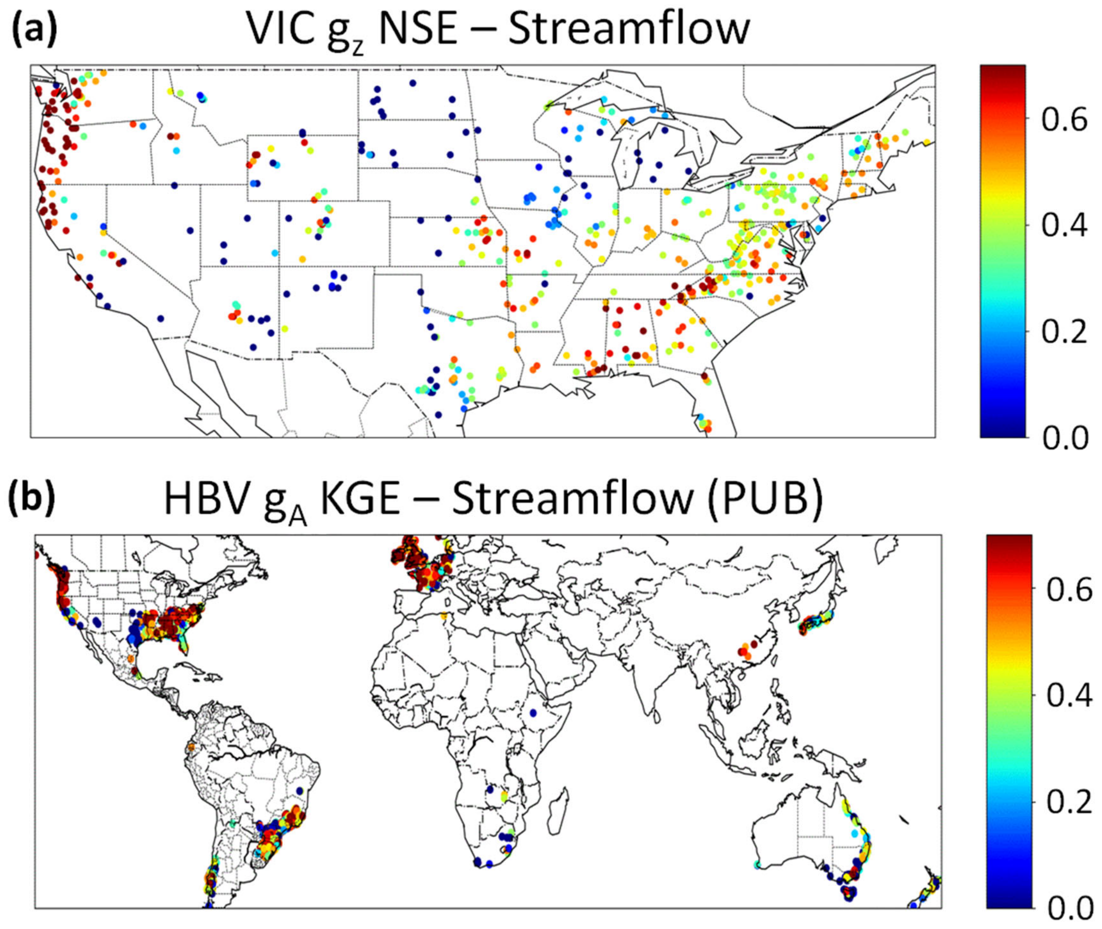

Figure S4. *Locations of basin outlets for (a) the Catchment Attributes and Meteorology for Large-Sample Studies (CAMELS). Color represents Nash Sutcliffe Model Efficiency coefficient (NSE) when using parameters estimated by* g_z *for the Variable Infiltration Capacity (VIC) hydrologic model; (b) The temperate group of the global headwater catchment database from Beck et al., 2020. Colors represent metric Kling-Gupta model efficiency coefficient (KGE) when using parameters estimated by* g_A *for the HBV hydrologic model.*

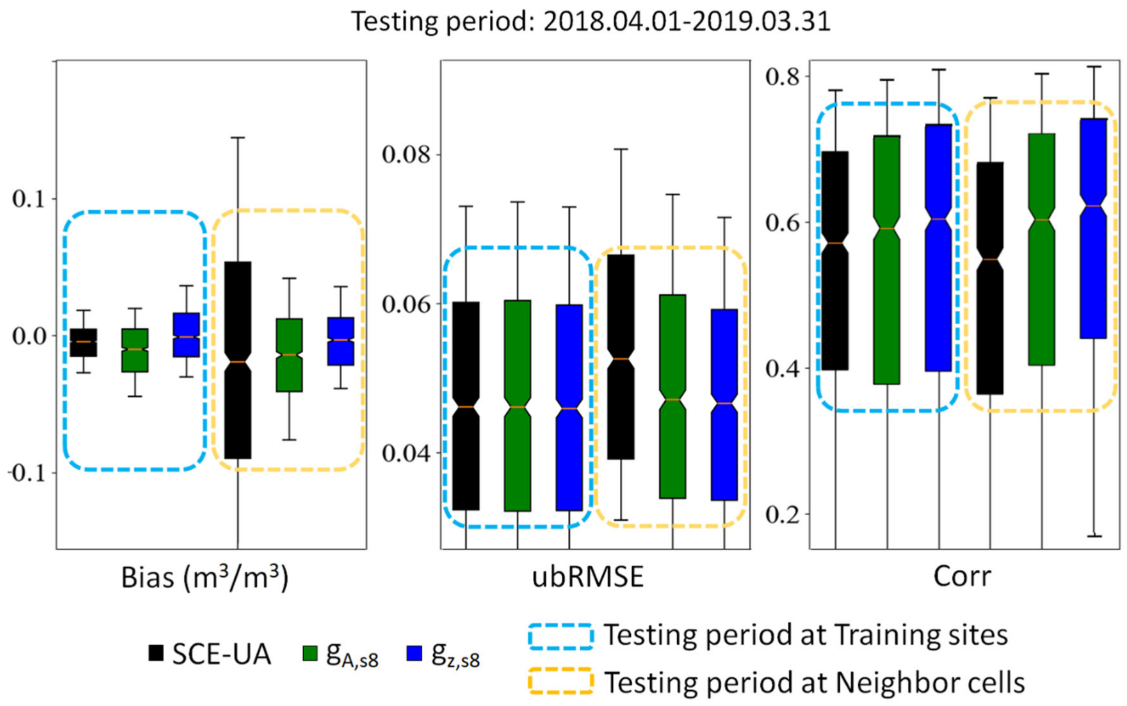

Figure S5. *Performance of SMAP soil moisture simulations for a different year than Figure 3 (2018-2019). Boxplots summarize metrics from gridcells for temporal generalization (evaluated on the training locations over the test period, blue dashed box) and spatial generalization (orange dashed box) tests at $1/8^2$ sampling density (one gridcell from an 8x8 patch, see illustration in Supplementary Figure S1) for SCE-UA and dPL options g_Z and g_A. In the spatial generalization test, we sampled at $1/8^2$ sampling density for training and evaluated the parameters' performance on a neighbor 3 rows to the north and 3 columns to the east from each of the training gridcells, over the test period. The boxplots' lower whisker, lower box edge, middle line, upper box edge and upper whisker represent 10th, 25th, 50th, 75th, and 90th percentiles, respectively. This figure shows that the relative advantages over SCE-UA remain very similar, i.e., SCE-UA deteriorated from training to test sites (at neighbor cells) while dPL had no deterioration for the test sites.*

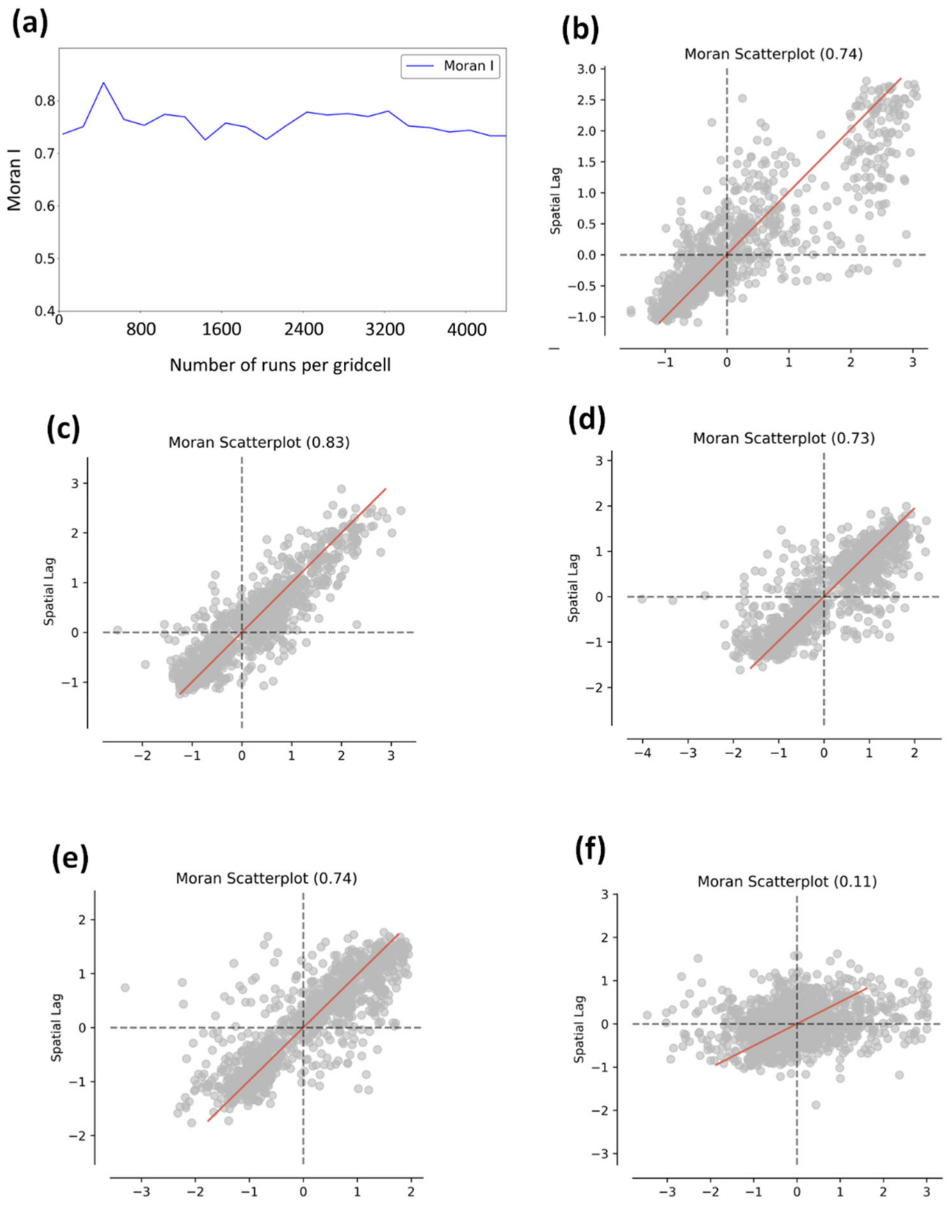

Figure S6. *Spatial autocorrelation of INFILT. (a) Moran's I values for INFILT from dPL as a function of the number of forward runs during training. Moran's I characterizes global spatial autocorrelation, and higher values indicate higher autocorrelation. (b)-(e) Moran scatterplots for dPL option* g_z *using 40, 400, 2000, and 4000 of runs per gridcell, respectively; (f) Moran scatterplot of SCE-UA at the end of optimization. The red line represents the best linear fit to the scatter plot.*

Table S1. *Mean and standard deviation (std) of CONUS-scale evaluation metrics of the ensemble (with different random seeds) for default NLDAS-2 parameters, SCE-UA-derived parameters, and dPL-derived parameters at 1/8^2 sampling density. NLDAS-2 does not have an ensemble so the std is absent.*

Metric	Parameter source	SMAP (mean ± std)	SMAP spatial extrapolation (mean ± std)
CONUS-median Bias	NLDAS-2 s8	0.045	-
	SCE-UA s8	-0.0012 ± 0.0001	-0.00103 ± 0.0014
	dPL gz s8	0.0016 ± 0.0026	0.00002 ± 0.0026
	dPL gA s8	-0.0011 ± 0.0019	-0.0017 ± 0.0027
CONUS-median Correlation	NLDAS-2 s8	0.5292	-
	SCE-UA s8	0.5891 ± 0.0025	0.5905 ± 0.0027
	dPL gz s8	0.6000 ± 0.0318	0.6170 ± 0.023
	dPL gA s8	0.5907 ± 0.0221	0.6020 ± 0.01258
